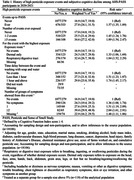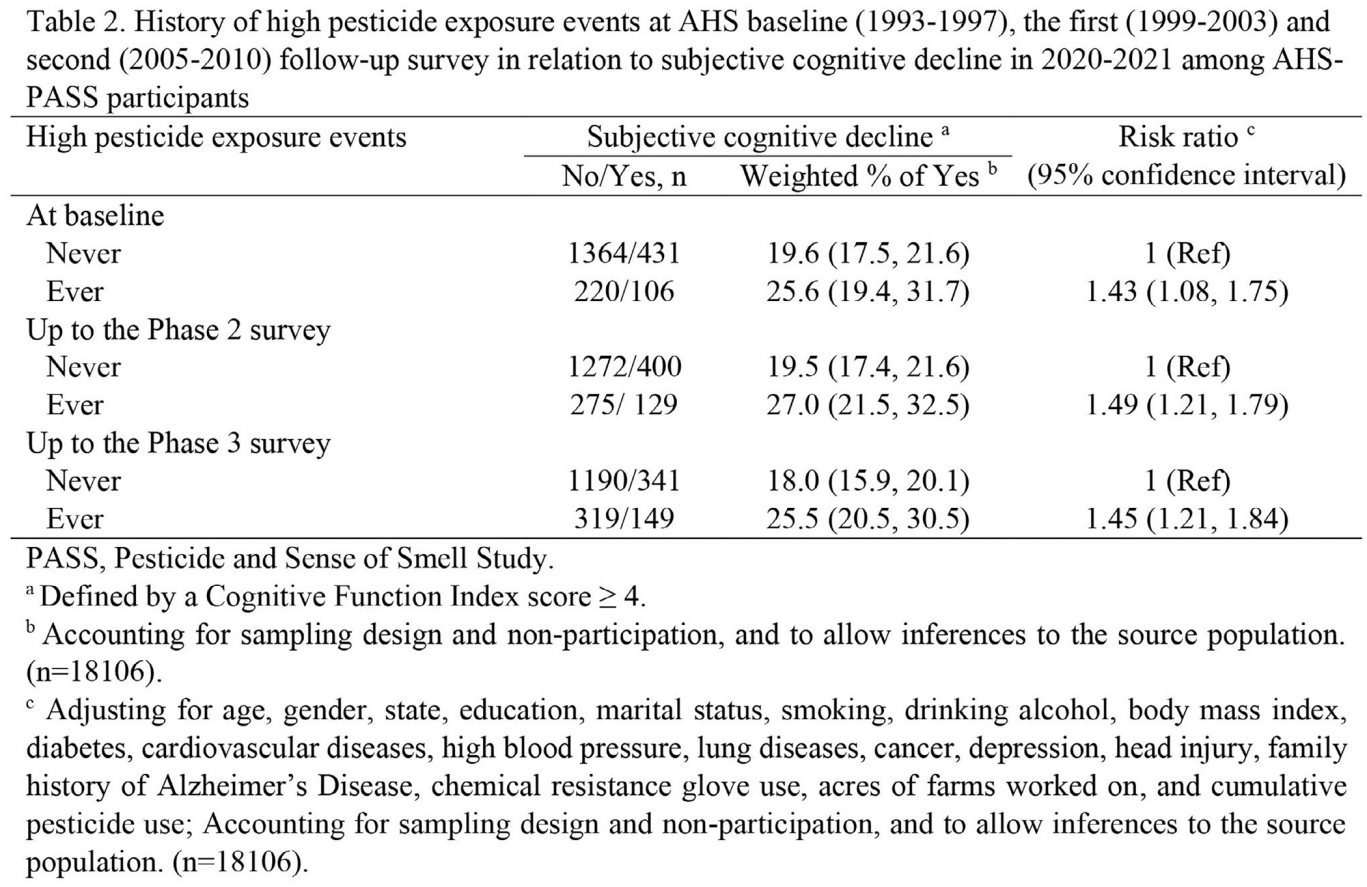# High Pesticide Exposure Events and Subjective Cognitive Decline among U.S. Farmers

**DOI:** 10.1002/alz.088003

**Published:** 2025-01-09

**Authors:** Yaqun Yuan, Brenda L Plassman, Shengfang Song, Zhehui Luo, Christine G Parks, Julie Fleenor, Corrine Madsen, Heather MacDonald, Scott Davis, Chenxi Li, Jonathan N Hofmann, Laura E. Beane Freeman, Dale P. Sandler, Honglei Chen

**Affiliations:** ^1^ Michigan State University, East Lansing, MI USA; ^2^ Duke University, Durham, NC USA; ^3^ National Institute of Environmental Health Sciences, Research Triangle Park, NC USA; ^4^ Duke University Medical Center, Durham, NC USA; ^5^ Duke University ‐ Joseph and Kathleen Bryan Alzheimer’s Disease Research Center, Durham, NC USA; ^6^ National Cancer Institude, Rockville, MD USA; ^7^ National Institute of Environmental Health Sciences, Durham, NC USA

## Abstract

**Background:**

Pesticide exposure may contribute to cognitive decline, but empirical evidence is limited. We examined high pesticide exposure events (HPEE) in relation to subjective cognitive decline among farmers in the Pesticide and Sense of Smell Study (PASS), a sub‐cohort of the Agricultural Health Study (AHS).

**Method:**

This analysis included 2365 predominantly white male farmers from Iowa and North Carolina (aged 70±10 years) who enrolled in AHS from 1993‐1997 and participated in PASS in 2020‐2021. Subjective cognitive decline was assessed in the PASS sub‐cohort using the 14‐item Cognitive Function Instrument (CFI), and significant decline was defined as a CFI score ≥ 4. HPEEs were assessed in 1993‐1997, 1999‐2003, and 2005‐2010 in the AHS cohort and in 2020‐2021 in PASS. We estimated the association of HPEE on SCD using a doubly robust, weighted least‐square method, accounting for study design, sampling, non‐response, and multiple covariates, including demographics, lifestyle factors, and major chronic diseases.

**Result:**

A history of HPEE was associated with significant subjective cognitive decline across all HPEE assessments in the AHS and PASS. Using the HPEE reported in PASS as an example, compared with those who never had any event, the risk ratio (RR) for those who reported HPEE was 1.57 (95% confidence interval: 1.35, 1.89). The association appeared stronger for more HPEE events (RR: 1.89 vs. 1.45 for ≥3 vs. 1‐2 events), longer delay in washing with soap and water (RR: 1.74 vs. 1.51 for waiting >1 vs. ≤ 1 hour), HPEE exposure via the respiratory/digestive tract (RR: 1.84 vs. 1.33 for dermal contact only), HPEE with subsequent symptoms (2.04 vs. 1.36 for ≥2 vs. no symptoms). Data on HPEE exposure to specific pesticides are limited, but we found significant associations of HPEE with SCD for alachlor, atrazine, 2,4‐D, and glyphosate.

**Conclusion:**

This study provides preliminary evidence that high pesticide exposure may contribute to subjective cognitive decline among older farmers.